# Exploring antigenic variation in autoimmune endocrinopathy

**DOI:** 10.3389/fimmu.2025.1561455

**Published:** 2025-02-28

**Authors:** Maria Mavridou, Simon H. Pearce

**Affiliations:** ^1^ Translational and Clinical Research Institute, Newcastle University, BioMedicine West, Newcastle-upon-Tyne, United Kingdom; ^2^ Endocrine Unit, Royal Victoria Infirmary, Newcastle-upon-Tyne, United Kingdom

**Keywords:** immunogenetics, autoimmune thyroid disease, thymus, central tolerance, Graves’ disease, Addison’s disease, type 1 diabetes

## Abstract

Autoimmune disorders develop owing to a misdirected immune response against self-antigen. Genetic studies have revealed that numerous variants in genes encoding immune system proteins are associated with the development of autoimmunity. Indeed, many of these genetic variants in key immune receptors or transcription factors are common in the pathogenesis of several different autoimmune conditions. In contrast, the proclivity to develop autoimmunity to any specific target organ or tissue is under-researched. This has particular relevance to autoimmune endocrine conditions, where organ-specific involvement is the rule. Genetic polymorphisms in the genes encoding the targets of autoimmune responses have been shown to be associated with predisposition to several autoimmune diseases, including type 1 diabetes, autoimmune thyroid disease and Addison’s disease. Mechanistically, variations leading to decreased intrathymic expression, overexpression, different localisation, alternative splicing or post-translational modifications can interfere in the tolerance induction process. This review will summarise the different ways genetic variations in certain genes encoding endocrine-specific antigens (INS, TSHR, TPO, CYP21A2, PIT-1) may predispose to different autoimmune endocrine conditions.

## Introduction

Autoimmune endocrinopathies are, almost by definition, organ-specific autoimmune diseases whereby one specific hormone-secreting gland or tissue becomes the target of the immune response leading to immune-mediated destruction and, over time, clinical hormonal deficiency (1–3). Only Graves’ disease (GD) deviates from this paradigm with stimulatory antibodies rather than a destructive immune response leading to organ hyperactivity and frequent clinical manifestations outside the primary organ of involvement (i.e. Graves’ orbitopathy). These disorders are mostly inherited as complex genetic traits with a multigenic basis, that is variations in numerous genes (typically 50 or more identified to date) each contribute a small degree towards the proclivity to clinical autoimmune disease (2, 4). Perhaps unsurprisingly, it has been more straightforward to identify genetic variations in immune-related genes that contribute to multiple autoimmune disorders, than to identify those disease or organ-specific variations. Many of these immune-related variants contribute to the susceptibility to more than one autoimmune condition, including both endocrine conditions and additional non-endocrine, organ-specific disorders (1–4). Some of the variants involved in several conditions are briefly reviewed in the next section.

Whilst the common autoimmune endocrinopathies are inherited as complex multigenic traits, there are two Mendelian conditions which provide relevant insights into mechanisms of immune tolerance. The first condition is the APECED (Autoimmune polyendocrinopathy, candidiasis and ectodermal dystrophy)/APS1 (autoimmune polyglandular type 1) syndrome, which is most frequently owing to loss of function mutations in both alleles of the AutoImmune REgulator (AIRE) gene (5). AIRE is expressed in thymic epithelial cells where it controls expression of transcripts relevant in thymic T cell selection. Defective AIRE function abrogates expression of certain self-peptides in antigen-presenting cells which is essential for educating the developing thymic T cell repertoire and leads to failure to delete T cells expressing potentially autoreactive T cell receptors. APS1 is characterised by high penetrance (70-90%) of autoimmune Addison’s disease (AAD, autoimmune primary adrenal insufficiency) and autoimmune hypoparathyroidism (5). Both of these conditions are rare outside the context of APS1, with a prevalence of one in 8,000 in the sporadic population for AAD, showing the critical role of central thymic tolerance in this condition.

More recent findings have also highlighted the contribution of common genetic variants in the *AIRE* gene to the susceptibility of AAD in a Swedish cohort. The minor alleles of four intronic strongly linked SNPs, rs9983695, rs2075875, rs2075876, and rs6220374, have been shown to have significantly lower frequency in Swedish AAD patients (approximately 4%) compared to healthy controls (ranging from 10% to 11%) (6). Although none of these variants overlap with known transcription factor binding sites, their strong linkage suggests they may play a regulatory role in AIRE’s function. This suggests that thymic antigen presentation is not just important in Addison’s disease associated with APS1, but perhaps unsurprisingly with sporadic AAD as well (6).

The role of AIRE gene variants in APS1 contrasts to the IPEX (Immune dysregulation, polyendocrinopathy, enteropathy, X- linked) syndrome, caused by defects in the *FOXP3* gene, encoding the critical transcription factor for development of regulatory T cells (7). IPEX is X-linked, giving rise to autoimmune diabetes before the age of 1yr in around 50% of affected boys, and signalling failure of peripheral tolerance as the primary mechanism. Thus, these 2 monogenic disorders demonstrate that genetic variants can impact the establishment and maintenance of immune tolerance via different mechanisms including on central T cell selection or by influencing peripheral regulatory lymphocyte function (5, 7).

## Brief review of immune system genetic variation in endocrine autoimmunity

Genome-wide association studies (GWAS) on patient cohorts with autoimmune endocrine disorders have revealed that most of the gene variants currently associated with autoimmunity encode proteins that have a role in the antigen recognition, and lymphocyte activation and inactivation pathways (3, 8). Whilst the focus of this review concerns gene variants that confer organ-specificity to the disease, that is the antigen or target-organ specific gene variants, we first make a brief review of the variants in immune system genes that have well-established roles in endocrine autoimmunity, with many of these variants contributing to disease risk in multiple different conditions (**Table 1**).

**Table 1:** Immune system genes linked to autoimmune endocrinopathies.

Locus	Gene	Disease
6p21	HLA class II	T1D, GD, HT, AAD
1p13	PTPN22	T1D, GD
2q33	CTLA4	T1D, GD, AAD
6q15	BACH2	T1D, GD, AAD,
10p15	IL2RA	T1D, GD, AAD

Genes involved in the immune system regulation with a role in the development of endocrine autoimmune disorders and their loci. T1D, Type 1 Diabetes; GD, Graves’ Disease; HT, Hashimoto’s Thyroiditis; AAD, Autoimmune Addison’s Disease.

The locus that has the strongest effect in the predisposition to most autoimmune disorders is the MHC, encoding the human leukocyte antigen (HLA) molecules. This locus spans a large interval on the short arm of chromosome 6, with numerous different HLA genes, each with many different alleles which may either confer risk or protection from autoimmunity. Most studies have identified associations of HLA class II alleles and certain HLA-DRB1-DQA1-DQB1 haplotypes with autoimmune endocrine disorders, implying the essential role of binding and presentation of endocrine antigens to CD4+ T cells in the pathogenesis of particular diseases. It is thought that variants in DR and DQ genes encode proteins that might bind self-peptides with lower affinity, potentially inadequate for the central elimination of autoreactive T cells resulting in impaired tolerance (8, 9). An alternative scenario suggests that certain DR and DQ proteins communicate inefficiently with the TCRs on Tregs leading to the generation of a Treg population that is unable to control autoreactive T cells. Additionally, variations in HLA class I genes have been also linked with susceptibility to autoimmunity. However, the strong Linkage Disequilibrium (LD), a term used to describe the non-random association between alleles of two or more genetic loci, between HLA class I and class II loci and the abundance of alleles often poses a difficulty in the identification of linked alleles having independent association with a phenotype (9, 10).

*The PTPN22* gene encodes the Lymphoid-tyrosine phosphatase (LYP) which is implicated not only in T and B cell signalling cascades, but also in natural killer (NK) cell and myeloid cell pathways. LYP acts by blocking T cell activation as it dephosphorylates CSK kinase and stops the downstream signal transduction. The rs2476601*T allele which leads to the Arg620*Trp substitution has been associated with autoimmune disorders including T1D and GD, with the variant having also been linked with relapse of GD after antithyroid treatment (11, 12). The Arg620*Trp variant is located in a highly conserved point in the P1 motif of the catalytic domain of LYP, suggesting the potential to directly affect phosphatase activity (13). The exact underlying mechanism is unclear as some studies have shown that this variant results in an LYP with loss of function that cannot inhibit T cell activation, whilst others suggest that the variant produces an LYP with gain of function and elevated inhibitory effect on T cell signalling pathway. The relevance of these investigations might be limited, though, as several of these functional studies have been conducted on the ortholog of LYP in mice, which shares 90% similarity in the catalytic and 60% similarity in the non-catalytic domains with the human homolog (1, 3, 8, 10, 14–17). More recent studies, however, suggest that the role of the substitution might be different depending on the cellular context in which the protein is expressed, or the location of the protein, implying that LYP might have a multifunctional role in the autoimmunity development (18). In particular, the variant might have a gain of function effect leading to an even stronger blocking of TCR signalling, and a subsequent diminished negative selection of autoreactive T cells in the thymus, and a reduced response to the BCR signal, and consequently hyper-responsive B cell populations. However, Zhang *et al*. have shown that in Jurkat T cells transduced with plasmids carrying the mutated LYP, and in PBMC obtained from rheumatoid arthritis (RA) patients homozygous for the risk allele, the Arg620*Trp substitution makes the protein more susceptible to intracellular degradation and therefore the variant leads to weaker inhibitory effects (19). Additionally, the variant leads to an LYP with loss of function in myeloid cells as it exhibits reduced interaction with TRAF3 and reduced amounts of TLR-induced type I IFNs (16–18, 20–22). These results suggest that there are pleiotropic effects with a gain of function phenotype centrally, along with loss of function effect in peripheral immune cells and, even more interestingly, different regulatory effects in lymphocytes than in myeloid cells (16, 17, 19, 23–25).

The Cytotoxic T-Lymphocyte Associated Protein 4 (CTLA4) is encoded by the *CTLA4* gene and is an inhibitory co-stimulatory receptor which binds to B7 molecules on the surface of antigen presenting cells, and consequently, downregulates their activation. Amongst the polymorphisms which have been associated with autoimmune disorders, the variant rs231775*G on the exon 1, and an (AT)_n_ repeat in the 3’-untranslated region (UTR) of the *CTLA4* gene have been linked to T1D and AAD. Both variants are associated with decreased expression of *CTLA4*, reducing levels of inhibitory signalling and, therefore, unchecked T cell activation. In addition, the frequency of rs3087243*G allele on the 3’-UTR of *CTLA4* gene is higher in GD and Hashimoto's thyroiditis (HT) patients than in healthy controls, with the allele also being associated with a reduction on the beneficial effects of anti-thyroid treatment in a Polish cohort with GD (26–29). The importance of the molecule can be understood by the fact that people with null *CTLA4* alleles develop immunodeficiency as a form of an autosomal dominant immune dysregulation syndrome, characterised by hypogammaglobulinemia and recurrent infections with several tissues being affected, whilst *CTLA4*-Knockout (*CTLA4*-KO) mice develop fatal lymphoproliferative disease in the first weeks of life (30).

BACH2 transcription factor (BTB and CNC Homology 1, Basic Leucine Zipper Transcription factor 2) is expressed predominantly in B lymphocytes and plays a vital role in regulating CD4+ T-cell differentiation. Its functions include repressing effector CD T-cell lineages (Th1, Th2, and Th17), and it is crucial for the formation of regulatory T cells. The intronic BACH2 variant rs3757247*T has been associated with susceptibility to AAD as the frequency of the risk T allele is significantly higher in UK AAD patients compared to controls (31). The frequency of the risk allele has been also found higher in Polish patients with autoimmune polyendocrine syndromes (APS) (*and in 6 Japanese T1D patients* (32)*)* compared to healthy individuals (33). Additionally, a recent study revealed a correlation between the risk T allele and the existence of circulating autoantibodies against thyroid peroxidase (TPO) in first-degree relatives of (Polish) AAD patients (34).

rs3757247 is in complete LD with the intronic rs11755527, rs72928017 and rs72928038 variants, with rs11755527 having been linked to susceptibility to T1D in northern European (35) and Pakistani populations (36), however no association was found in a Brazilian population (37). rs3757247 and rs11755527, along with another intronic SNP, rs2474619, have been associated with GD in a Chinese Han population with rs2474619 determined as the most disease-associated variant (11, 38). rs72928038*A allele has been associated with risk of development of T1D as the variant leads to lower expression of *BACH2* in multiple cell types, but mainly in T cells (39). The risk allele has been shown to impede the binding of the ETS1 transcription factor leading to decreased enhancer activity (39, 40). rs72928038*A has been also correlated with GD and HT in a UK cohort (11, 41). Thus, the exact molecular mechanisms by which the genetic variants affect gene regulation and the unravelling of the causative variant remain unidentified and require further investigation.

Interleukin-2 (IL-2) is a key driver of T cell maintenance, proliferation, and development. Polymorphisms on the gene encoding the alpha subunit of the IL-2 receptor (CD25) have been associated with T1D, GD *(and AAD although with contradictory results in different populations: UK-Norway)*. Amongst those, T1D patients carrying the intronic rs2104286*C allele have lower *CD25* expression on their naïve CD4+ T cells, but higher levels of soluble CD25. Thus, the variant could lead to an impaired IL-2 signalling transduction and lower responsiveness of T cells to IL-2 effects. Such a decrease in IL-2 signalling might have a sequential impact on *FOXP3* expression and Tregs activity, with the protective minor allele of the variant rs12722495 probably acting in an opposite way (10, 14, 42–44).

Identification of susceptibility loci is a complex process as the genes involved in disease pathogenesis are often in LD. Furthermore, power is frequently limited by cohort size, meaning that reproducibility is a key issue. Furthermore, genetic heterogeneity between affected patients of different ethnicities is not unexpected, complicating the interpretation of some studies (3, 8, 10, 14, 15). A deep understanding of the genetic – epigenetic interactions in combination with environmental triggering events (viral or bacterial infections, alteration of gut and oral microbiome) that might have additional effects is required for an accurate unravelling of the multiple pathogenesis mechanisms of autoimmunity (45).

## Role of target-organ genes in endocrine autoimmunity

### Insulin gene variation in Type 1 Diabetes

Type 1 Diabetes Mellitus (T1D) is a chronic disease which is characterised by the autoimmune destruction of the insulin secreting β cells in the pancreatic islets by T lymphocytes (CTLs), leading to insulin deficiency. Similar to other autoimmune disorders, it is believed that a combination of numerous genetic variants, each with a small effect, along with environmental factors are implicated in the pathogenesis (46). These various susceptibility loci have been labelled as *IDDM1, IDDM2* etc. in chronological order of discovery, with *IDDM1* residing in the MHC on chromosome 6p21. As well as being the hormone that is deficient in T1D, insulin autoantibodies and T cell responses against insulin peptides are also central to its pathogenesis (46, 47). However, autoantibodies to other islet cell protein targets including glutamic acid decarboxylase (GAD) and insulinoma-associated 2 (IA-2) are highly prevalent and may predate the onset of T1D by many years.

One of the non-HLA loci involved in the disease susceptibility is a variable number of tandem repeats (VNTR) region upstream of the start codon of the insulin gene, *INS*, on chromosome 11. The region, which has also been known as the *IDDM2* locus, is a minisatellite as it consists of repeats of a 14bp sequence with variable length; alleles have been distinguished by repeat number with class I alleles having a shorter length, whilst classes II and III have more repeats of the sequence (47–54). Studies in T1D patients and healthy controls have shown that *IDDM2* class I alleles are associated with the disease susceptibility, with class I homozygosity increasing the risk for the disease development by 2-5 times (53). On the contrary, class III alleles, even in heterozygosity, have been shown to be dominantly protective.

Studies on the mRNA levels of insulin have shown that class I alleles are associated with increased *INS* mRNA levels in the pancreas compared to class III alleles (47, 50, 52). However, the opposite effect has been observed in human thymus, where class I alleles have been linked to decreased *INS* expression compared to class III alleles. Therefore, it is believed that the mechanism by which *IDDM2* class I and III alleles can regulate the predisposition to T1D is by controlling the thymic expression levels of the insulin gene and its subsequent epitope presentation by HLA molecules to maturing T cells (50). With lower thymic expression of insulin peptides in VNTR class I carriers, some INS-reactive TCR-carrying T cells escape clonal deletion leading to autoreactivity and ultimately T1D. Experiments in which insulin gene copy numbers were manipulated in knockout (KO) mice, confirm that thymic *INS* expression is strongly genetically determined and that animals with low or absent thymic insulin mRNA had autoreactive peripheral T cell clones against insulin. Conversely, mice with high thymic insulin expression were protected from insulin autoreactivity (55).

A genetic study involving detailed phenotyping of German families with children at risk of developing T1D confirmed that the short, class I VNTR was associated with early onset diabetes. Interestingly, individuals with the dominantly protective class III VNTR had a lower risk of developing insulin autoantibodies, but had similar rates of GAD and IA2 antibodies, supporting that this variation at the *INS* locus was acting directly to determine the prevalence of insulin autoreactivity (56).

Although it might not be intuitively obvious that variations leading to high expression of an autoantigen might protect against autoimmune disease, these experiments in both human and rodent demonstrated for the first time that thymic gene expression forms a critical tolerogenic mechanism that may be affected by naturally occurring human genetic variation.

## Graves’ disease

Graves’ disease (GD) is a common autoimmune disorder of the thyroid gland characterised by the continuous activation of the thyroid stimulating hormone receptor (TSHR) by autoantibodies, leading to hyperthyroidism. Although the reason why these TSH receptor stimulating antibodies, known as TRAb, occur is unknown, it is believed that, again, an interaction of environmental and genetic factors might trigger their appearance (57–60). TRAb are central to disease pathogenesis and circulating TRAb concentrations correlate tightly with disease severity and clinical outcome (61). Unlike T1D which has an approximately equal gender distribution, GD affects 5-8 times more women than men. The TSHR is a G protein-coupled receptor, consisting of an extracellular A subunit, bound to a transmembrane domain (B) subunit by a flexible hinge region. The immunodominant T cell and B cell epitopes reside within the extracellular A-subunit. Soluble, circulating A subunits are shed from the cell membrane and found in the circulation, where they may consist of several homo-oligomeric forms (62).

Amongst the genetic loci associated with GD susceptibility, HLA-*DRB1*, *PTPN22*, *CTLA-4*, there is also the *TSHR* gene which encodes the main autoantigen. Studies on the non-coding regions of the *TSHR*, showed that the A allele in the intronic rs179247 SNP is strongly associated with disease susceptibility, whilst it is also linked to the earlier onset of the disease (57, 59, 60, 63). Analysis of the *TSHR* expression revealed that although the polymorphism does not have any effect on the expression levels in the thyroid gland, it can influence the mRNA levels in the thymus. More specifically, individuals homozygous for the high-risk A allele have been shown to have lower *TSHR* expression levels in their thymus compared to people carrying the protective G allele, with G/G homozygous showing the higher *TSHR* expression (57, 59). Thus, it has been suggested that, with similarities to the *INS* situation for T1D, a gene variant that regulates (mRNA or protein) expression quantitatively in the thymus, can lead to the disease development through inadequate recognition of ‘autoreactive’ T cells and breakdown of negative T cell selection.

An alternative mechanism which has also been suggested, though, links the high-risk rs179247*A allele with alternative splicing of the *TSHR* gene. Studies have shown that people carrying the rs179247*A allele have lower levels of the full-length *TSHR* mRNA, and increased levels of two alternative shorter truncated transcripts in the thyroid gland. These alternative forms lack the transmembrane and the hinge region and therefore lead to the production of soluble TSHR isoforms, similar to the A-subunit part of the receptor (59, 63–72). These secreted TSHR forms can be presented and contribute to the loss of peripheral tolerance (57, 59). Additionally, elevated levels of these soluble isoforms compared to the full-length receptor might result in increased production of autoantibodies against TSHR, as it has been shown that the A-subunit of the receptor is the main target of the autoantibodies and also promotes the affinity maturation of the B cells producing thyroid-stimulating antibodies (68, 72).

However, a more recent study measuring the expression levels of the different isoforms has shown that the disease-associated genotype was associated with higher levels of the alternative splice variants in the thymus, and not in the thyroid, implying that these might have a role in the induction of the central tolerance in the thymus instead (57). In contrast to the case of *INS*, it has been proposed that expression of non-immunodominant TSHR epitopes in thymus could have a role in inducing tolerance (57).

**Figure 1:**
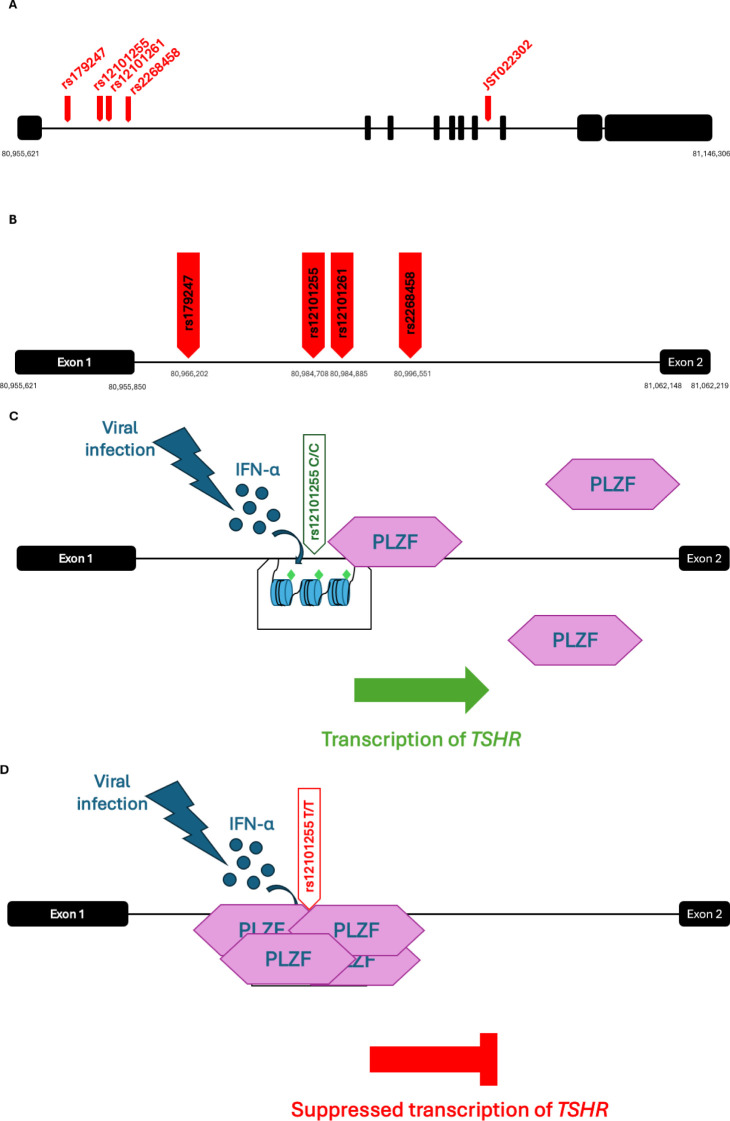
**(A)** Schematic representation of the GD-associated SNPs on the *TSHR* gene. The 10 exons are represented as black squares and SNPs as red arrows. The minor allele of the polymorphism JST022302 in intron 7 has been associated with the disease in a Japanese cohort (64, 79, 80) **(B)** Schematic representation of the GD-associated SNPs in intron 1 of the *TSHR* gene. **(C, D)** Effects of environmental events on individuals with genetic predisposition to GD; IFN-α is secreted in response to a viral infection event and induces monomethylation at the Lysine 4 of Histone 3 (H3K4me1) (represented as green diamond shapes) in the intronic region of TSHR containing the disease-associated variants. The transcription repressor PLZF binds with a stronger affinity in people homozygous for the high-risk rs12101261*T allele **(D)**, than in individuals homozygous for the protective C allele **(C)**. Thus, a viral infection can cause a reduction in the expression of *TSHR* in the thymus of people carrying the high-risk allele of the variant **(D)**, and therefore lead to a decreased presentation of the protein epitopes to T cells. GD, Graves' Disease; TSHR, thyroid stimulating hormone receptor; IFN-α, Interferon alfa; PLZF, promyelocytic leukemia zinc finger transcription factor. [Image created in PowerPoint and adapted from (59, 73, 130)].

rs12101255, another SNP linked to the disease susceptibility, in intron 1 of the *TSHR* gene, is in complete LD with its adjacent rs12101261. The two polymorphisms were found to overlap with a region of monomethylated histone 3 lysine 4 (H3K4me1), a chromatin modification induced by IFN-α, during a viral infection (59, 60, 63). The region has been shown to be critical for the *TSHR* expression as it is the binding site of the promyelocytic leukaemia zinc finger (PLZF) transcription factor. PLZF binds with a stronger affinity in individuals homozygous for the high-risk rs12101261*T allele, and is correlated with lower intrathymic expression of *TSHR*, compared to people homozygous for the protective allele C (**Figure 1**) (59, 60, 63). Thus, an environmental event, such as a viral infection, might influence the expression of an autoantigen gene in individuals carrying the high-risk allele of this variant. Decreased expression of the *TSHR* in the thymus, induced by IFN-α production, in a susceptible individual could trigger autoimmunity development through failure to delete autoreactive T cells (59, 60, 63, 73–76).

The first SNP which has been associated with the disease susceptibility in Caucasians (UK and Polish populations), rs2268458 (77), is located in the same 40-kb region of intron 1 of *TSHR* gene together with rs179247, rs12101255 and rs12101261, and all of them are in moderate LD posing additional difficulty in the dissection of the causative SNP. However, logistic regression analysis on the most highly associated SNPs, rs179247 and rs12101255, performed by a group studying the association of their minor alleles with elevated expression of the truncated isoforms of the receptor in UK and Polish Caucasians, revealed that the rs12101255 might drive the association of the nearby variants (64, 78, 79), although rs179247 had previously been shown as the variant with the strongest association with the disease in UK GD patients (64).

Meta-analysis of different studies showed that there is a level of consistency in the results amongst Caucasian and Asian populations. Although the high-risk alleles in intron 1 were confirmed in a Spanish GD cohort (63), interestingly, a study in Japanese GD patients in 2005 revealed the existence of SNPs in introns 7 and 8 of *TSHR* that were also associated with the disease, and this association was confirmed when these variants were tested in a UK GD cohort (64, 79, 80). More recent studies performed by the China Consortium for the Genetics of Autoimmune Thyroid Disease confirmed the association of the intron 1 variants (rs12101261) with the disease susceptibility in Chinese patients, but did not show significant association of the intron 7 variants that had been previously detected in a Japanese cohort (76). These results indicate that although there may be complex LD patterns that vary between ethnicities, variants on intron 1 of the *TSHR* play an important role on the disease susceptibility in Caucasian and Asian populations (64, 78–80).

## Hashimoto’s thyroiditis

Hashimoto’s thyroiditis (HT), the most common autoimmune disorder, causes destruction of the thyroid gland by lymphocytic infiltration and leads to hypothyroidism. The presence of circulating autoantibodies against the main thyroid antigens, thyroid peroxidase (TPO) and thyroglobulin (Tg) is crucial for the diagnosis, but the mechanism behind their occurrence is not yet known. Twin studies have shown that genetic susceptibility plays a role in the disease development, but environmental events, such as stress and iodine consumption, also contribute to disease onset (81–83).

TPO is an enzyme involved in the biosynthesis of the thyroid hormones T4 and T3 and is encoded by the *TPO* gene located on chromosome 2. It is uniquely expressed in thyroid. Polymorphisms in the *TPO* gene have been associated with both the development of the disease and the levels of circulating autoantibodies. The rs2071400 SNP in the promoter of *TPO* has been shown to have a potential role in the disease pathogenesis. Studies have shown that the prevalence of the rs2071400*T allele is significantly higher in people with hypothyroidism than in healthy controls. In addition, homozygosity for the rs2071400*T variant is significantly more frequent in individuals with hypothyroidism compared to healthy people (81, 84–88). These findings suggest that the rs2071400*T allele is a risk factor for the development of hypothyroidism. Another variant which has been linked with disease predisposition (in an Indian population) is the missense variant, rs732609, in exon 12 of the *TPO gene*, with rs732609*C occurring more frequently in patients with hypothyroidism than in controls, and therefore conferring risk for the disease onset. Substitution of the A allele with C, leads to the change of Threonine725 to Proline, which is predicted to be a structurally tolerated substitution. However, this may still alter expression of *TPO* or its enzymatic activity (85, 88–90).

**Figure 2:**
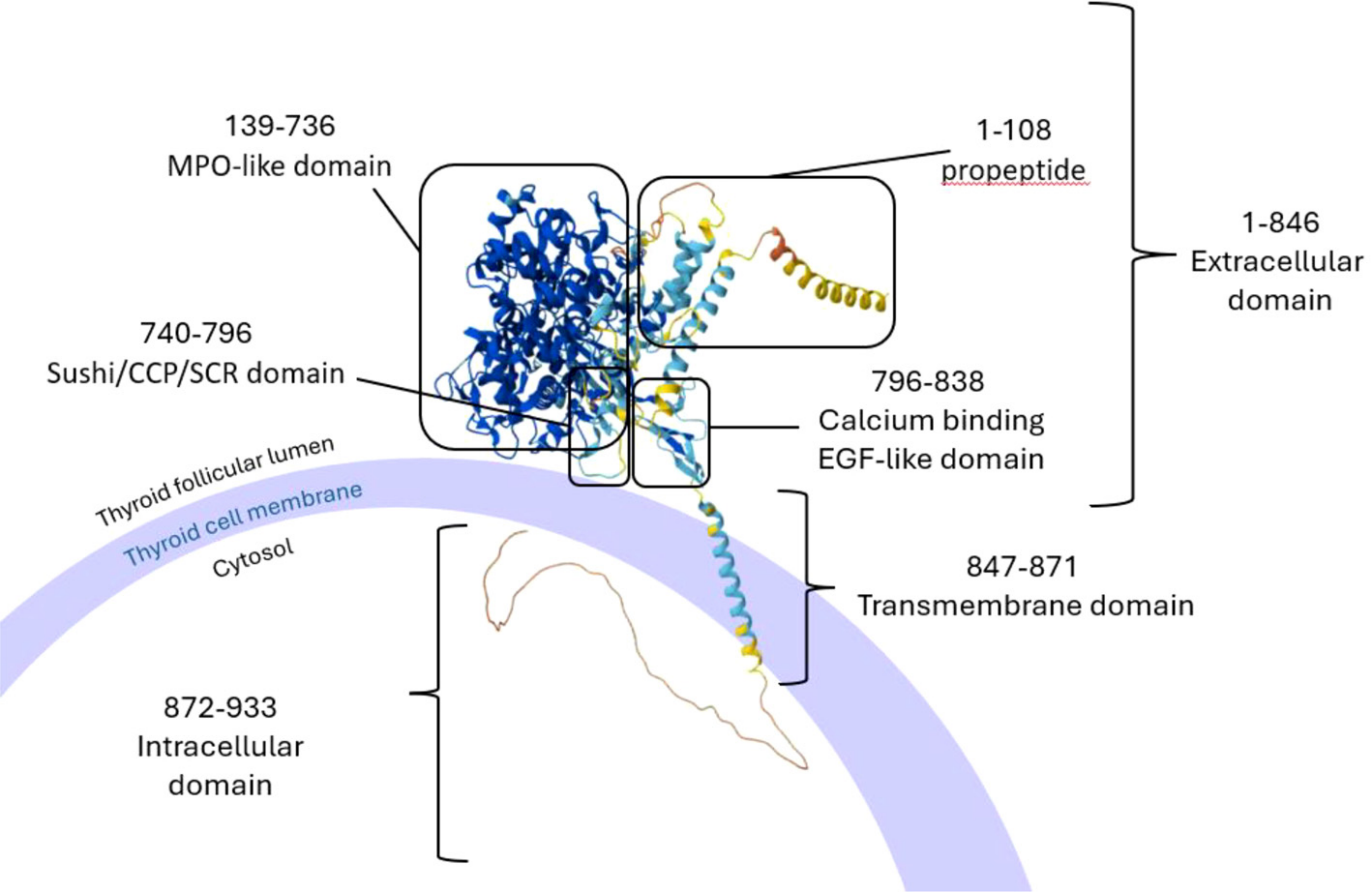
Schematic representation of the structure of TPO with its domains as it is bound on the membrane of thyroid cells. Image created in PowerPoint with the structure of the protein created by AlphaFold and the domains analysed by InterPro (result/InterProScan/iprscan5-R20241108-164444-0398-33458951-p1m/Overview). AlphaFold produces a per-residue model confidence score (pLDDT) between 0 and 100. Some regions below 50 pLDDT may be unstructured in isolation. Very high (pLDDT > 90) is shown in dark blue, high (90 > pLDDT > 70) in cyan blue, low (70 > pLDDT > 50) in yellow and very low (pLDDT < 50) in orange. TPO, thyroid peroxidase, MPO; myeloperoxidase; CCP, complement control protein; SCR, short consensus repeat; EGF, epidermal growth factor. Image adapted from (91, 92, 131).

The large extracellular domain of TPO is structurally homologous to the enzyme myeloperoxidase (MPO) and the threonine 725 (Thr725) residue is located within this MPO-like domain of TPO (**Figure 2**). This domain is crucial for the activity of the enzyme, and therefore substitution of Threonine with Proline might have an impact on the enzyme’s peroxidase activity. The residue is also close to an immunodominant B cell epitope of TPO, consisting of nine amino acids, and is recognised by the well-characterised monoclonal TPO antibody, MAb-47. Studies have shown that the MAb-47 recognises the native structure of human TPO, and thus there is a possibility that the nearby residues play a crucial role in the maintenance of the conformational structure of the epitope (91–94).

Similar to sodium iodide symporter (*NIS*), and *TSHR*, expression of *TPO* has been detected in the Hassall’s corpuscles of human thymus tissue, although it was found to be expressed at lower levels compared to other thyroid antigens like Tg and TSHR by a Korean study. The same study showed a correlation between the expression levels of TPO and age, as TPO expression in the thymus was shown to be increased with increasing age (95). Another study by Misharin *et al*. on *AIRE*-KO mice, which investigated the correlation between AIRE and the expression of thyroid antigens in the thymus, revealed that intrathymic *TPO* expression was absent in AIRE deficient animals, whereas *Tg* and *TSHR* were still expressed, albeit at reduced levels (96, 97).

Thyroglobulin (Tg), the precursor molecule of T3 and T4 hormones, is encoded by the *Tg* gene which is located on chromosome 8. Although association studies have linked this locus with predisposition to autoimmune thyroid disorders in general, studies on HT patients have shown that the rs180195*G allele in the promoter region of the *Tg* gene is significantly more frequent in HT patients than in healthy individuals (75, 87, 98, 99). Substitution of the wild-type A allele with G, leads to an increase in the activity of the *Tg* promoter through the modification of the transcriptional activator interferon regulatory factor 1 (IRF-1) binding site. IRF-1 is activated in response to IFNAR signalling pathway and controls the expression of IFN-induced genes (75). It is possible, then, that an increase in IFN levels/activity as a protective mechanism against a potential infection could cause an increased expression of the *Tg* gene and therefore elevated presentation of the Tg epitopes. This finding can possibly explain the development of thyroid autoimmunity in people who have previously received IFN-α treatment and poses another example of genetic-epigenetic and environmental factors interaction for the induction of autoimmunity (75, 82, 87).

**Figure 3:**
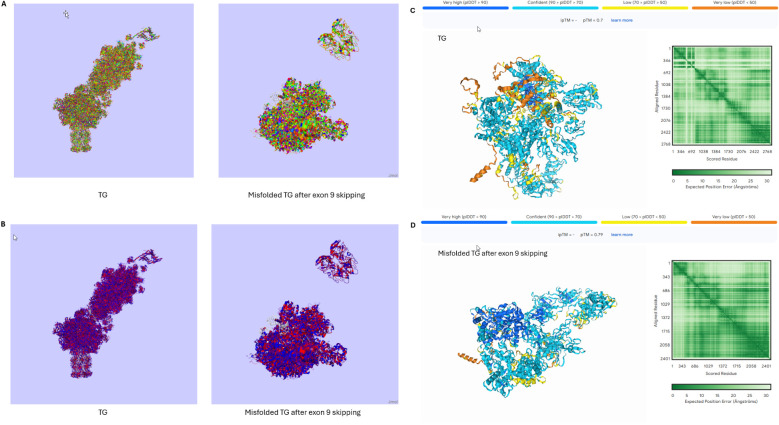
3D structures of the normal TG and the misfolded TG after the skipping of exon 9 (introduced by the rare variant c.1076-1G > C). **(A)** Taylor colour schemes highlighting the amino acids based on their physicochemical properties. Figures created in Jalview 2.11.4.1. **(B)** Hydrophobicity colour schemes highlighting the amino acids based on their hydrophobicity. Figures created in Jalview 2.11.4.1. **(C, D)** Prediction of the 3D structures of the normal TG **(C)** and the misfolded TG protein **(D)** based on their amino acid sequences. Figures created in Alphafold server. AlphaFold produces a per-residue model confidence score (pLDDT) between 0 and 100. Some regions below 50 pLDDT may be unstructured in isolation. Very high (pLDDT > 90) is shown in dark blue, high (90 > pLDDT > 70) in cyan blue, low (70 > pLDDT > 50) in yellow and very low (pLDDT < 50) in orange. TG, Thyroglobulin.

The rare splice site variant, c.1076-1G > C, which leads to the deletion of the whole of exon 9 of the *Tg* gene, has been found in all affected members of a family with early HT onset (100, 101). The shorter transcript, produced by the variant, might lead to the synthesis of a misfolded protein. Misfolding would either lead to an immunogenic isoform or expose different epitopes of the peptide sequence compared to the wild-type Tg (**Figure 3**). If the misfolded isoform is bound to MHC class II molecules, these alternative epitopes would not be recognised as ‘self’ by T cells, as they are not presented under normal conditions, and therefore activate an immune response; whilst if they manage to avoid intracellular degradation, they might also modify the level of post-translational modifications of the Tg, such as iodination (100–103). Although we cannot be sure about the mechanism causing this phenotype, the presence of autoantibodies against the Tg and the TPO in these patients implies that there is ongoing immunogenicity. Additionally, it is worth considering that any condition leading to increased thyroid cell turnover might predispose to autoimmunity. Co-existing autoimmune thyroiditis has been well-documented in conditions such as resistance to thyroid hormone beta (RTHβ) and Pendred’s syndrome, both of which are associated with goiter through distinct mechanisms. In RTHβ, mutations in the thyroid hormone receptor beta gene lead to impaired feedback regulation of the hypothalamic-pituitary-thyroid axis and persistent TSH stimulation of the thyroid with higher cell turnover and goiter, which may expose cryptic antigens and increase susceptibility to autoimmune thyroid diseases such as HT and GD (104–106). Similarly, Pendred’s syndrome is characterised by mutations in the *SLC26A4* gene and causes thyroid dyshormonogenesis and deafness. Chronic TSH stimulation therefore leads to thyroid enlargement (goitre) and potentially in susceptible individuals increased thyroid antigen expression (106–109). Thus, loss of function Tg variants are known to impair thyroid hormone synthesis leading to congenital dyshormonogenetic goiter, and hypothyroidism. Genetic variants leading to less profoundly reduced Tg function (e.g. c.1076-1G > C), might be associated with inefficient rather than absent thyroid hormone synthesis and could lead to autoimmunity by means of increased TSH and thyrocyte turnover, goiter and higher thyroid antigen expression.

Studies on mice with experimental autoimmune thyroiditis (EAT), a murine model of HT, have proposed the co-existence of two T cell populations; the Tg-reactive T cells and the natural CD4+CD25+ nTregs that are specific for the Tg (110). These studies have shown that continued Tg epitope presentation is essential in order to maintain a population of nTregs which are critical for the control of Tg-reactive T cells in the periphery. Decreased levels of Tg in the circulation or presentation of alternative epitopes might disturb the balance required for the nTregs maintenance, and therefore lead to ‘escaped’ Tg-reactive T cells (81, 110, 111). This mechanism of peripheral tolerance contrasts to that demonstrated for insulin gene expression in the thymus.

## Autoimmune Addison’s disease

Autoimmune Addison’s disease (AAD) is a rare autoimmune endocrine condition characterised by the destruction of the adrenal cortex. Autoantibodies against the steroid 21-hydroxylase (21-OHase) enzyme are detected in most patients, with the underlying mechanism for their production, though, remaining unclear (1, 8, 112). Given the fact that 21-OHase is an intracellular enzyme and unlikely to be accessible to circulating autoantibodies, their direct pathogenic role has never been demonstrated. However, the existence of antigen-specific T cells in the blood of AAD patients has been demonstrated and the presence of CD4+ and CD8+ T cells bearing 21-OHase-specific TCRs against two immunodominant 21-OHase epitopes has been confirmed, demonstrating the central contribution of Cytotoxic T cells to disease progression (8, 113, 114). Like in other autoimmune endocrine disorders, studies on twins and family members of AAD patients have shown that genetics play an essential role in the disease development. However, the rise in cases the recent years implies that environmental events, such as viral infections and stress, might also be involved (1, 5, 8, 115).

**Figure 4:**
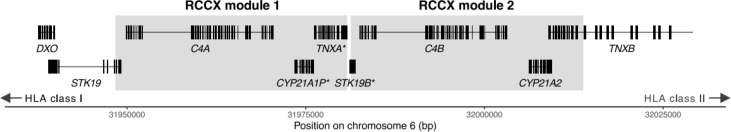
Schematic representation of the most common form of the RCCX cassette in healthy individuals, with pseudogenes noted with an asterisk. Figure adapted by **Figure 1A** of Lundtoft *et al.* (122).

21-OHase is encoded by the *CYP21A2* gene, which is located in the MHC class III region on chromosome 6, and mutations on this gene are the commonest cause for Congenital Adrenal Hyperplasia (CAH) (1, 116, 117). The MHC class III region is located in the middle of the MHC gene cluster, flanked by the MHC class II (*HLA-DR, DQ*) genes on one side and MHC class I (HLA-A -C) on the other. Very near the CYP21A2 locus, there is an adjacent highly homologous pseudogene, *CYP21A1P*, which cannot produce a functional enzyme due to an 8-bp deletion in the exon 3 which results in a truncated protein (1, 118). Both the location and the high sequence identity of the two genes contribute to recombination events which are considered the main reason for the deleterious mutations accumulating in the *CYP21A2* gene that lead to CAH (118). The two genes, *CYP21A2* and *CYP21A1P*, belong to a repeated genomic cassette, called the RCCX module. Apart from the two CYP21 genes, in its most frequent form in Caucasians, the locus contains the serine/threonine kinase 19 (*STK19*) gene, the tenascin-X (*TNX*) gene, the complement 4 A (*C4A*) and B(*C4B*) genes, and the pseudogenes *STK19B* and *TNXA*. Non-allelic homologous recombination (NAHR) events in the locus alter the module structure and cause gene conversions and deletions increasing the complexity of the region (118) (**Figure 4**). Sequencing of the *CYP21A2* gene from AAD patients and healthy individuals showed five SNPs that occur more often in heterozygosity in AAD patients compared to controls (119). Three of them, rs61338903, rs6474 and rs6473, are located in the exonic sequence, whilst the other two, rs6467 and rs76565726, are intronic variants (119). The minor allele of variant rs6473 has been shown to regulate the transcriptional repressor CTCF binding site and has been associated with downregulation of *HLA-C* and *HLA-DRB1* expression (120). In addition, AAD patients with 21-OHase antibodies were more likely to carry these variants in a heterozygous state. However, regression analysis revealed that all variants were in LD with HLA-DRB1*03:01, *04:01 and *04:04 alleles which are associated with high-risk for AAD (119, 121). In contrast, the rs6472*C allele was found to be protective against the disease, and independent from the HLA-DRB1 locus, with a significantly lower frequency in AAD patients compared to controls (119). Thus, it remains unclear whether the primary association at this locus is with HLA-DR alleles or whether variants in *CYP21A2* itself could contribute to AAD susceptibility.

Analysis of the copy number variation of the CYP21 genes showed different patterns for the functional *CYP21A2* and the pseudogene *CYP21A1P* between healthy controls and AAD patients. Copy numbers of *CYP21A2* were similar between AAD patients and healthy people, with 95% of the individuals carrying 2 copies of the gene. Interestingly, the *CYP21A1P* showed larger variation ranging from 0 to 5 copies, with AAD patients revealed as more likely to have lower copy numbers than the healthy controls, with each ‘lost’ copy being associated with a 3.4-fold odds ratio for disease (122). Examination of the copy number variation of the Complement-4 A (*C4A*) and B (*C4B*) genes, which are located in the same RCCX cassette, showed that there is a stronger correlation of *CYP21A1P* variation with *C4A* than with *C4B*, however lower copy numbers of the C4A gene were slightly more associated with AAD compared to those of *CYP21A1P* (122). Nevertheless, lower copies of both *CYP21A1P* and *C4A* are in close LD with the high risk HLA-DRB1*03:01 allele, as the SNPs described above, and therefore it remains unclear whether they might have an independent effect (119, 122, 123). It is not immediately obvious why copy number or expression of *CYP21A1P*, a pseudogene, should be relevant for disease pathogenesis. However, we speculate that AAD patients have reduced copy number or absence of *CYP21A1P*, because thymic expression of this pseudogene could underly central tolerance, in a similar way to that found for insulin gene variation.

## Anti–pituitary-specific transcriptional factor-1 syndrome (Anti-PIT-1 hypophysitis)

Immune-mediated pituitary dysfunction is a rare condition, either involving the cells of the anterior pituitary alone, or involving the posterior pituitary to produce an associated vasopressin deficiency, known as infundibulohypophysitis. Anti-PIT-1 hypophysitis is a rare but recently recognised autoimmune pituitary disease, which is characterised by growth hormone (GH), prolactin (PRL), and thyroid stimulating hormone (TSH) deficiency (124–127). PIT-1 is an important transcription factor for the control of GH, PRL and TSH expression in the somatotroph, lactotroph and thyrotroph cells of the anterior pituitary gland respectively, and mutations on the *PIT-1* gene cause congenital GH, PRL, and TSH deficiency. The presence of not only circulating anti-PIT-1 antibodies, but also CD8+ T cells in the pituitary gland of patients with anti-PIT-1 hypophysitis, indicate another example of a tissue-specific autoimmune disorder where CTLs are implicated in organ-specific cell damage (124, 125, 127–129). Thus far, the disease has only been found in the presence of thymoma or other types of neoplasms that have ectopic expression of the *PIT-1* gene (124). Although it is currently unclear what types of malignancies coexist with the disease, and in which proportion, the underlying mechanism involves ectopic expression and presentation of PIT-1. Thus, somatic mutations leading to ectopic expression of *PIT-1* in thymoma(s), or other malignancies, represents another mechanism by which dysregulation of antigenic gene expression can precipitate an organ-specific autoimmune endocrine disease.

## Perspective for other autoimmune conditions

Autoimmune endocrine disorders may be considered relatively unique amongst autoimmune conditions in that the clinical manifestations occur largely after a hormone-secreting cell-type is irreversibly damaged leading to hormonal deficiency. This contrasts to a disease such as systemic lupus erythematosus (SLE), where the nuclear antigens are ubiquitous in all cell types and damage causing some of the clinical presentations is mediated by immune-complex deposition. Thus, there are some striking differences in antigen distribution and it is easy to envisage how peripheral immune tolerance may have a more important role in systemic autoimmune diseases with less restricted antigen distribution/expression than for organ-specific ones. Even within the spectrum of autoimmune thyroid disease, it is notable that patients with APS1, a ‘pure thymic defect’ in tolerance (5), very rarely develop GD, but quite frequently develop HT. This may reflect that TSH receptor A-subunit is shed from the thyrocyte membrane (68) leading to degree of peripheral tolerance, whereas thyroid peroxidase has an apical (internal) membrane localisation and perhaps restricted access from ongoing immune surveillance. As mentioned above, role of antigen itself in autoimmunity is relatively neglected and further studies could cast important light on this subject.

## Conclusion

Genetic variation in organ-specific antigens remain understudied in autoimmunity in general. However, because of the discrete involvement of endocrine glands/tissues in autoimmune endocrinopathy, we are starting to glimpse potential antigen-specific mechanisms that underly disease proclivity for these conditions. Ectopic expression of many antigens in thymus may be a critical stage in T lymphocyte selection and under-expression may lead to failure to delete auto-reactive TCR-bearing T cell populations. Importantly, thymic gene/antigen expression may be regulated differently to that of the native tissue, leading to complexity in understanding the pathophysiological link. In contrast, continuous exposure of the peripheral immune system to endogenous antigen, such as Tg, may also provide an important mechanism for tolerance. We have much to learn in this area, but a fuller understanding of the antigenic stimulus and the detail of how this is involved in pathogenesis holds the promise of a tissue-specific solution rather than a general ‘immunosuppressive’ approach to therapy for autoimmunity.
